# Is proximal tibial tubercle osteotomy superior to distal tibial tubercle osteotomy for medial compartmental osteoarthritis? A meta-analysis

**DOI:** 10.1186/s13018-023-03725-5

**Published:** 2023-03-27

**Authors:** Min Song, Xiaodong Lin, Weichang Han, Jingyi Li, Wengang Liu

**Affiliations:** 1 Clinical Medical College of Acupuncture and Rehabilitation, Guangzhou University of Chinese Medicine, Guangzhou, China; 2 Fifth Clinical Medical School, Guangzhou University of Chinese Medicine, NO.60 Hengfu Road, Guangzhou, 510095 Guangdong China

**Keywords:** Distal tibial tubercle osteotomy, Proximal tibial tubercle osteotomy, High tibial osteotomy, Medial knee osteoarthritis, Meta-analysis

## Abstract

**Background:**

Open-wedge high tibial osteotomy (OWHTO) is commonly performed for the treatment of medial compartment knee osteoarthritis (KOA), and is classified into proximal tibial tubercle osteotomy (PTO) and distal tibial tubercle osteotomy (DTO). The PTO osteotomy point is generally located about 3–4 cm below the joint of the proximal tibia, and the osteotomy line points to the upper part of the proximal tibiofibular joint. The DTO osteotomy point is generally located about 0.5–1.0 cm below the tibial tubercle, and the osteotomy line points to the contralateral cortex. However, there is currently no consensus on which surgical technique is superior. The purpose of our study was to investigate which among the two is superior for medial KOA, with respect to knee joint parameters, clinical function, and complications.

**Methods:**

This study was conducted as per the PRISMA (Preferred Reporting Items for Systematic Reviews and Meta-Analyses) guidelines. The Cochrane Central Library, MEDLINE, Embase, PubMed, CNKI, and WanFang databases were systematically searched for trials comparing PTO and DTO in patients with medial compartment KOA, from inception until March 2022. The meta-analysis was conducted using RevMan 5.2 software. The Cochrane risk-of-bias tool was used to assess methodological quality. Statistical analysis was performed with Stata 12.0. Outcomes of interest included the Insall-Salvati index (ISI), Caton-Deschamps index (CDI), Blackburne-Peel index (BPI), posterior tibial slope (PTS), and the Hospital for Special Surgery (HSS) knee-rating scale.

**Results:**

A total of 15 retrospective studies (910 knees) were included. There were no significant differences in the age or sex of included patients. There was a significant difference in the ISI, CDI, BPI, and PTS between the two groups (all *p* ≤ 0.05). Further, DTO was associated with a significantly greater number of postoperative complications (*p* < 0.05) compared to PTO. However, there was no significant difference in the femorotibial angle (FTA), mechanical medial proximal tibial angle (mMPTA), and HSS knee score (all *p* > 0.05).

**Conclusions:**

Compared with DTO, PTO is associated with a greater incidence of postoperative patella baja and increased PTS, whereas DTO is associated with more postoperative complications. Nevertheless, both can significantly correct knee varus deformity and improve knee function; their early knee function scores are also similar.

*Trial Registration*. Prospective Register of Systematic Reviews (PROSPERO) registration number CRD42021284443.

## Background

Conventional medial open-wedge high tibial osteotomy (OWHTO) is commonly performed for the treatment of medial compartment knee osteoarthritis (KOA) with good clinical efficacy. Depending on the osteotomy position, OWHTO can be divided into proximal tibial tubercle osteotomy (PTO) and distal tibial tubercle osteotomy (DTO) [1, 2]. Recently, several studies have shown that PTO increases contact pressure in the patellofemoral (PF) joint, leads to patella baja, and increases the posterior tibial slope (PTS) [3–5]. The occurrence of patella baja can affect the biomechanics of the knee, causing anterior knee pain, patellofemoral arthritis, and reduced range of motion of the knee joint. Further, patella baja and increased PTS may make the revision of total knee arthroplasty difficult and affect subsequent rehabilitation [3, 6]. Studies have shown that about 20% of patients with high osteotomy require TKA after 10 years. Reduced patella height may increase the difficulty of knee replacement. DTO can avoid patella baja and patellar tendon, resulting in scar contraction. Strong internal fixation can improve the defect of long healing time, and has less influence on future joint replacement. Therefore, TKA after PTO is more difficult compare to DTO [7]. The progression of patellofemoral OA due to patella baja is reported as a particular complication related to PTO. In PTO, the tibial tubercle is attached to the distal tibia fragment by ascending osteotomy of the tibial tubercle; therefore, a gap opening at the transverse osteotomy may induce patella baja and a change in patella tracking [8]. It may result in lateral displacement of the tibial tubercle, which lead to an increase in contact pressure or adhesions of the patella ligament [9], and consequent cartilage degeneration in the patellofemoral joint. However, the cause of patellar arthritis after OWHTO is still inconclusive and needs further study. Kim et al. [10] in a study of 114 patients, found that the incidence of patellofemoral OA in the femoral trochlea was 41.2% 2 years after PTO, while that in the patellar articular surface was 21.9%. As per the existing literature, increased PTS may contribute to anterior cruciate ligament (ACL)-injuries, and may be associated with a higher risk of ACL reconstruction failure [11]. There are many disadvantages to DTO, such as the high likelihood of the screw being inserted into the tibial tubercle and penetrating the posterior tibial cortex, which may injure the popliteal neurovascular bundle. Owing to the irregular anatomy of the proximal tibia, the fixation screws for the tibial tubercle after PTO osteotomy need to be implanted from front to back, which further increases the risk of posterior vascular and nerve injury [12–14]. To the best of our knowledge, there is no relevant meta-analysis discussing the difference between PTO and DTO techniques in the treatment of medial compartment KOA. Therefore, we performed a meta-analysis of related clinical studies to determine whether PTO is superior to DTO in the treatment of medial compartmental KOA. The aim of the study was to assess whether DTO would change the patellar height (PH) and PTS, and result in improved clinical outcomes compared to PTO. The study hypothesis was that after DTO, (1) there is no changes in patellar position or (2) there is no increased PTS, and (3) there would be clinical improvement as objectively measured by the HSS score.

## Materials and methods

### Methodology

The meta-analysis was performed according to the Preferred Reporting Items for Systematic Reviews and the Guidelines for Meta-Analysis (PRISMA) [15]. We searched PubMed, Cochrane Library, Embase, MEDLINE, CNKI, and Wanfang for clinical studies comparing PTO and DTO in the treatment of unicompartmental osteoarthritis of the knee, from inception until March 2022, with no language constraints. RevMan 5.2(Review Manager (RevMan) [Computer program]. Version 5.2. Copenhagen: The Nordic Cochrane Centre, The Cochrane Collaboration, 2012.) software was used to carry out the meta-analysis. We optimized the search with the following keywords: (high tibial osteotomy OR open-wedge osteotomy OR open-wedge proximal tibial osteotomy OR open wedge high tibial osteotomy) and (proximal tibial tubercle osteotomy OR descending HTO OR supra-tubercle cut OR monoplanar, distal tibial tubercle osteotomy OR ascending HTO OR infra-tubercle cut OR biplanar). In addition, we examined the list of references in all the included papers to help identify studies that met the inclusion criteria.

### Criteria for inclusion and exclusion

The inclusion criteria were as follows: (1) research design: retrospective studies, prospective cohort studies, and randomized controlled trials, (2) participants: patients with OA in the medial compartment of the knee joint, (3) comparison of DTO and PTO outcomes, and (4) inclusion of the following indicators: PH of CDI or BP or ISI, femorotibial angle (FTA), mechanical medial proximal tibial angle (mMPTA), PTS, HSS knee score, and postoperative complications.

Exclusion criteria were (1) Reports on either DTO or PTO (2) non-clinical reports, (3) duplicate publications, (4) meeting proceedings, (5) studies with the above evaluation parameters not included.

### Data extraction

Data were collected by two researchers based on the same inclusion criteria, and were checked by a third researcher. Differences were resolved by mutual consensus. The corresponding authors were contacted when data or necessary supporting information was missing. The following data were collected: (1) study information (i.e., author, year of publication, and type of study), (2) study population information (i.e., age, sex), (3) surgery type and follow-up, and (4) principal outcomes (i.e., PH, FTA, mMPTA, PTS, HSS knee score, and complications. As for PH, it included the BPI, ISI, and CDI.

### Quality assessment

The risk of bias in the methodological quality of the RCTs was assessed using tools from the Cochrane Collaboration, collected by two independent researchers, and verified by a third researcher [16]. The evaluation included seven items: subject blinding, randomization, missing data, allocation concealment, outcome evaluator blinding, selective reporting, and other biases. Deviation risk was rated as "low", "high," or "uncertain". The nonrandomized studies were evaluated by the Risk of Bias in Non-Randomized Studies of Interventions (ROBINS-I) assessment tool [17]. Evaluation is mainly based on the following seven aspects: bias due to confounding, bias in the selection of participants, bias in the measurement of interventions, bias due to departures from intended interventions, bias due to missing data, bias in the measurement of outcomes, and bias in the selection of the reported result.

### Statistical analysis

The meta-analysis was performed by RevMan 5.2 software, provided by the Cochrane Collaboration. Heterogeneity tests were performed on the 95% confidence intervals (CIs). The mean (MD) and standard deviation (SD) were calculated for quantitative data, and the odds ratio (OR) with 95% CIs were calculated for count data. A *p* value less than 0.05 was considered statistically significant (*p* < 0.05). The Q test and *I*^*2*^ value were used to determine statistical heterogeneity between the included studies. *I*^2^ ≤ 50% indicated absence of statistical heterogeneity, and the fixed-effect model was adopted, while* I*^2^ ≥ 50% indicated statistical heterogeneity among the included studies, we used a model of random effect.

## Results

**Fig. 1:**
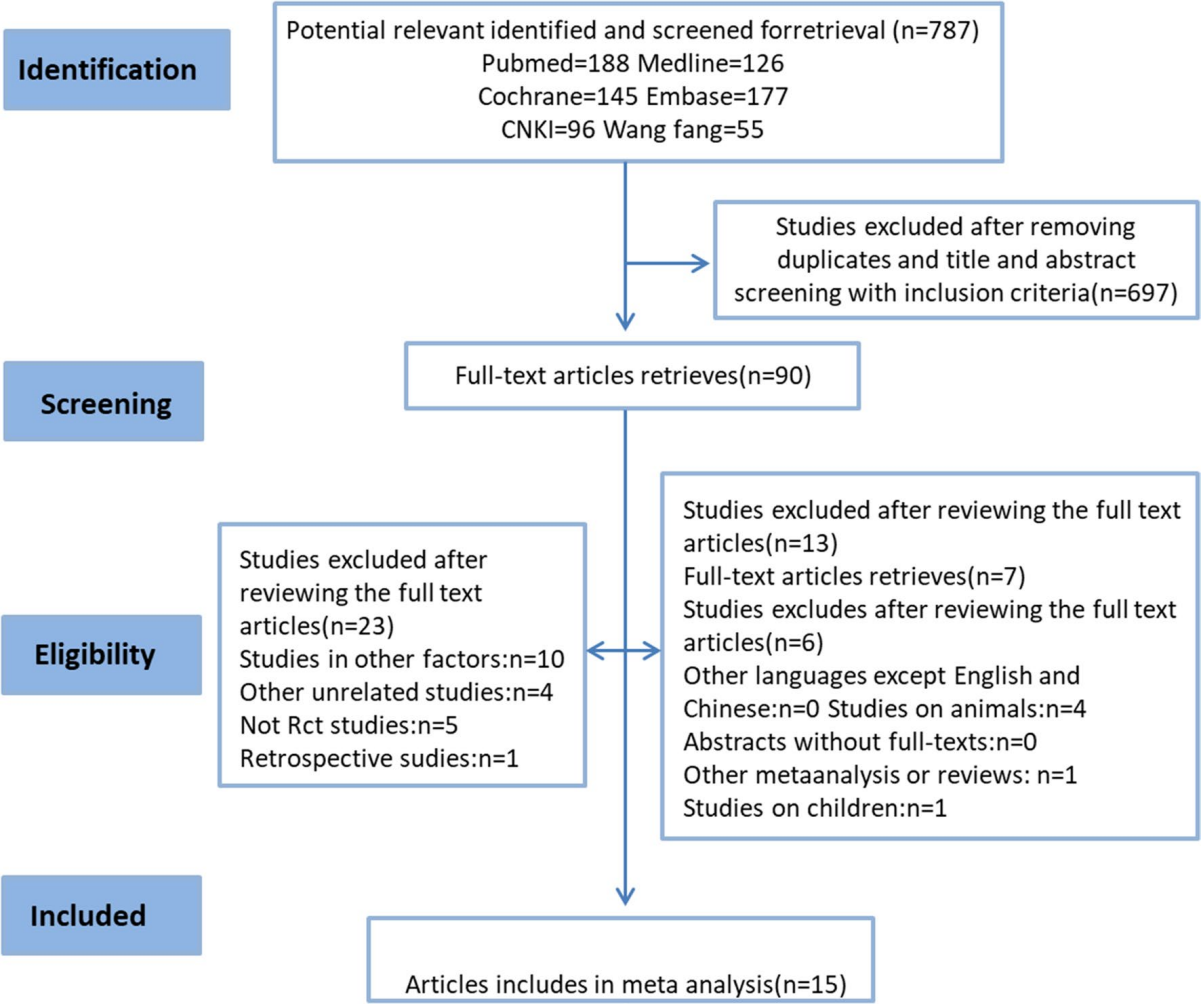
The flow chart of literature screening

After evaluation, 15 studies met the requirements for inclusion in this meta-analysis, providing a combined sample size of 910 knees (Fig. 1).

### Risk of bias assessment

All the 15 studies included were retrospective studies (i.e., nRCTs); therefore, were evaluated using the ROBINS-I assessment tool [17], as shown in Table 1. These studies were independently evaluated by two authors and disputes were resolved by a third author until consensus was reached.

**Table 1:** Methodological assessment according to ROBINS-I

nRCT Study = 15	Bias due to Confounding	Bias in Selection of Participants	Bias in Measurement of Interventions	Bias due to Departures from Intended Interventions	Bias due to Missing Data	Bias in Measurement of Outcomes	Bias in Selection of the Reported Result	Overall Bias
Elmalı et al. [18]	Low	Low	Low	Low	Low	No information	Low	Moderate
Krause et al. [20]	Low	Low	No information	Low	Low	Low	Low	No information
Hoon et al. [19]	Low	Moderate	Low	No information	Moderate	Low	Low	Low
Hinterwimmer et al. [29]	No information	Low	Low	Low	Low	Low	Low	Low
Longino et al. (2013)	Low	Low	No information	Low	No information	Low	No information	Low
Gooi et al. [25]	No information	No information	Critical	Low	Low	Low	Low	Low
Horikawa et al. [13]	No information	Low	Low	Low	Low	Low	Low	Low
Gaasbeek et al. [12]	Low	Low	Low	Moderate	Low	No information	Low	Low
Ogawa et al. [24]	Critical	No information	Low	Low	Low	Moderate	Low	Low
Liu et al. [23]	Low	Critical	Moderate	Moderate	No information	Low	No information	No information
Chen [21]	Low	No information	Low	Low	Low	Critical	Low	Low
Jianchun et al. [28]	Low	Critical	Low	Low	Low	No information	Low	Low
Zhang et al. [26]	Moderate	Low	No information	Low	Low	Low	Low	Low
Zhang et al. [27]	Low	Moderate	Moderate	Low	Low	Low	Critical	Low
Du et al. [22]	No information	No information	Low	Low	Low	Low	Low	Critical

### Outcome measure

#### Insall-Salvati index (ISI)

**Fig. 2:**
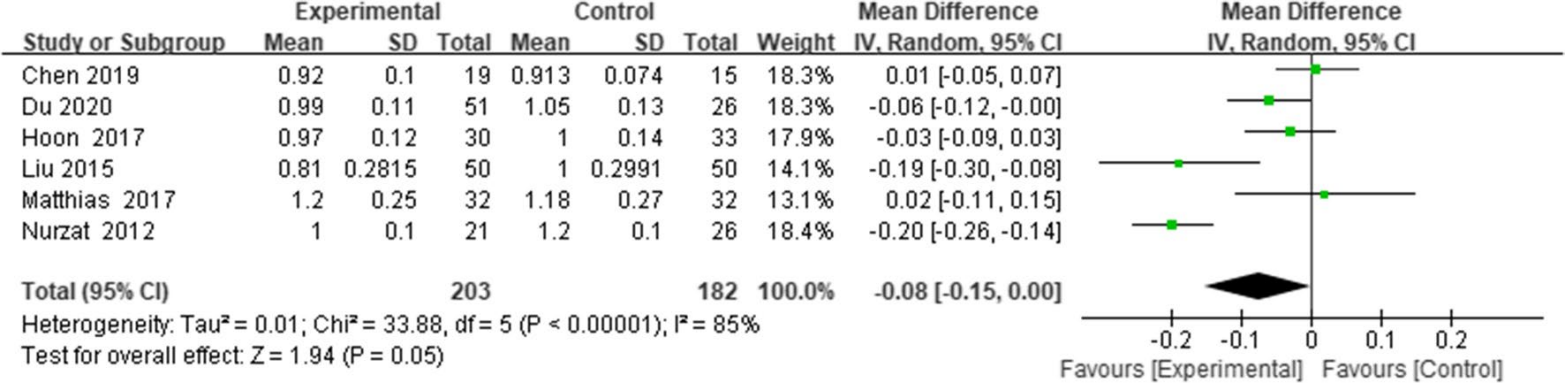
Forest plot of ISI

Six studies reported the ISI [18–23]; the combined results suggested that PTO decreased the ISI (weighted mean difference (WMD) = -0.08, 95% CI = (-0.15 to 0.00), *p* = 0.05, *I*^*2*^ = 85%) (Fig. 2).

#### Caton-Deschamps index (CDI)

**Fig. 3:**
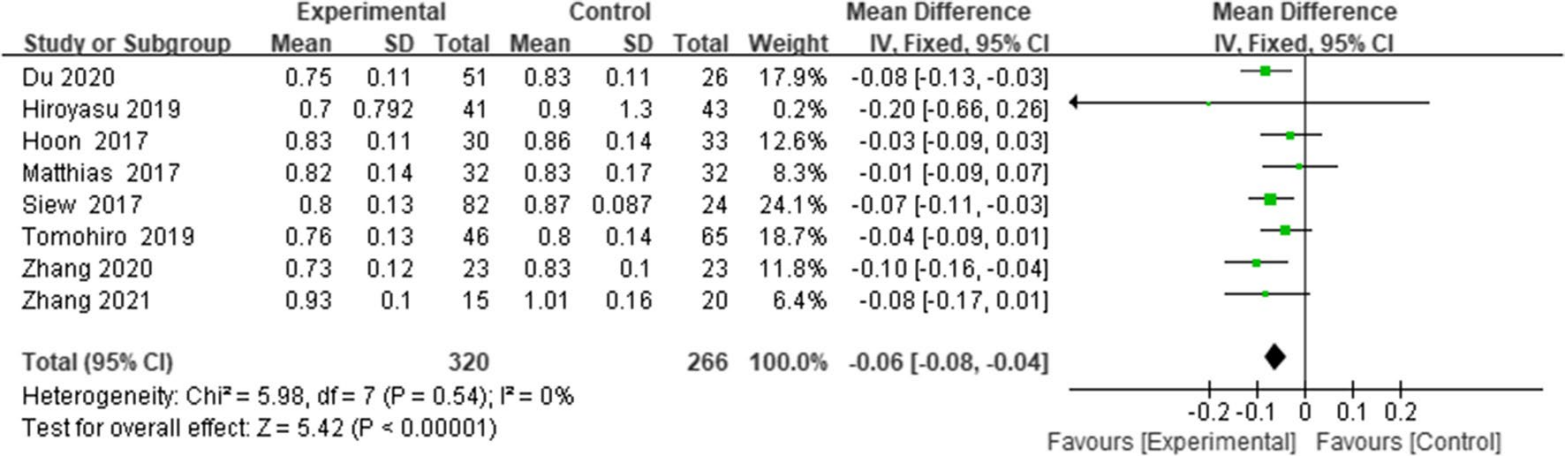
Forest plot of CDI

Eight studies [13, 19, 20, 22, 24–27] r reported the CDI; the combined results suggested that PTO decreased the CDI (WMD = -0.06, 95% CI = (-0.08 to -0.04),* p* < 0.00001,* I*^*2*^ = 0%) (Fig. 3).

#### Blackburne-Peel index (BPI)

**Fig. 4:**
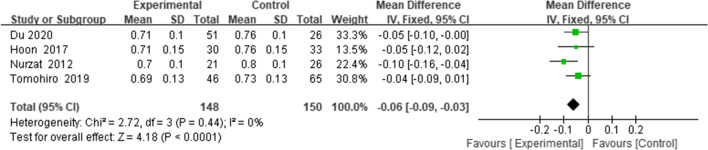
Forest plot of BPI

Four studies [13, 18, 19, 22] reported the BPI; the combined results suggested that PTO decreased the BPI (WMD = -0.06, 95% CI = (-0.09 to -0.03), *p* < 0.0001, *I*^*2*^ = 0%) (Fig. 4).

#### Posterior tibal slope (PTS)

**Fig. 5:**
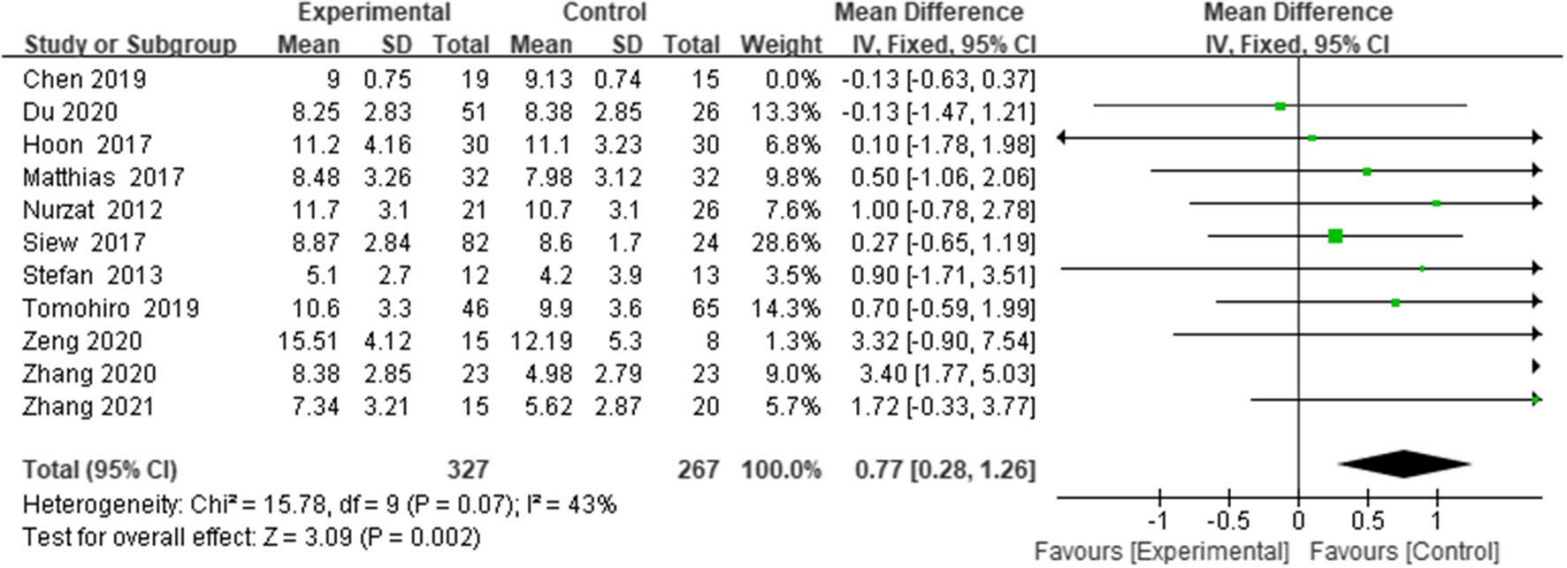
Forest plot of PTS

Eleven studies [13, 18–22, 25–28] reported the PTS. Since significant heterogeneity was noted among the study groups (*I*^*2*^ = 55%; MD = 0.33, 95% CI: -0.02–0.69, *p* = 0.01), the random-effects model was adopted. Sensitivity analysis found that the variance of PTS data in one study was not consistent with that in other studies, possibly due to its different measurement methods, and heterogeneity was reduced when this data was removed (*I*^*2*^ = 43%). The results showed that PTO increased PTS (WMD = 0.77, 95% CI = (0.28 to 1.26), *p* <  = 0.002, *I*^*2*^ = 43%) (Fig. 5).

#### Femoro tibal angle (FTA)

**Fig. 6:**
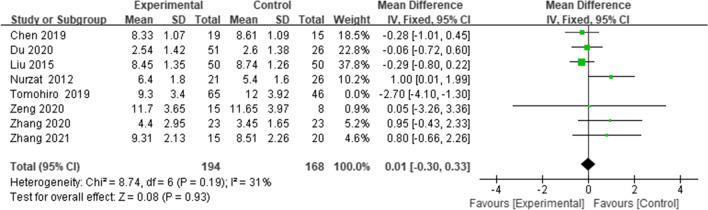
Forest plot of FTA

Eight studies [13, 18, 21–23, 26–28] reported the FTA; the results suggested no major difference in the FTA between the two groups (WMD = 0.01, 95% CI = (-0.30 to 0.33),* p* = 0.93, *I*^*2*^ = 31%) (Fig. 6).

#### Mechanical medial proximal tibial angle (mMPTA)

**Fig. 7:**
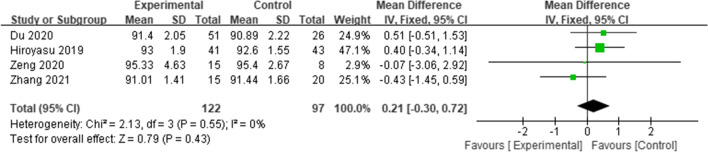
Forest plot of mMPTA

Four studies [22, 24, 27, 28] reported the mMPTA; the combined results showed no significant difference in the mMPTA between the two groups (WMD = 0.21, 95% CI = (-0.30 to 0.72),* p* = 0.43, *I*^*2*^ = 0%) (Fig. 7).

### HSS knee score

**Fig. 8:**
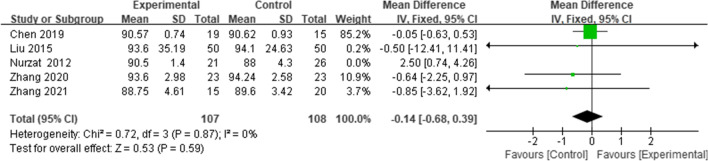
Forest plot of HSS knee score

Five studies [18, 21, 23, 26, 27] reported the HSS knee score; the combined results showed no significant difference in the HSS knee score between the two groups (WMD = -0.14, 95% CI = (-0.68 to 0.39), *p* = 0.59, *I*^*2*^ = 0%) (Fig. 8).

### Complications

**Fig. 9:**
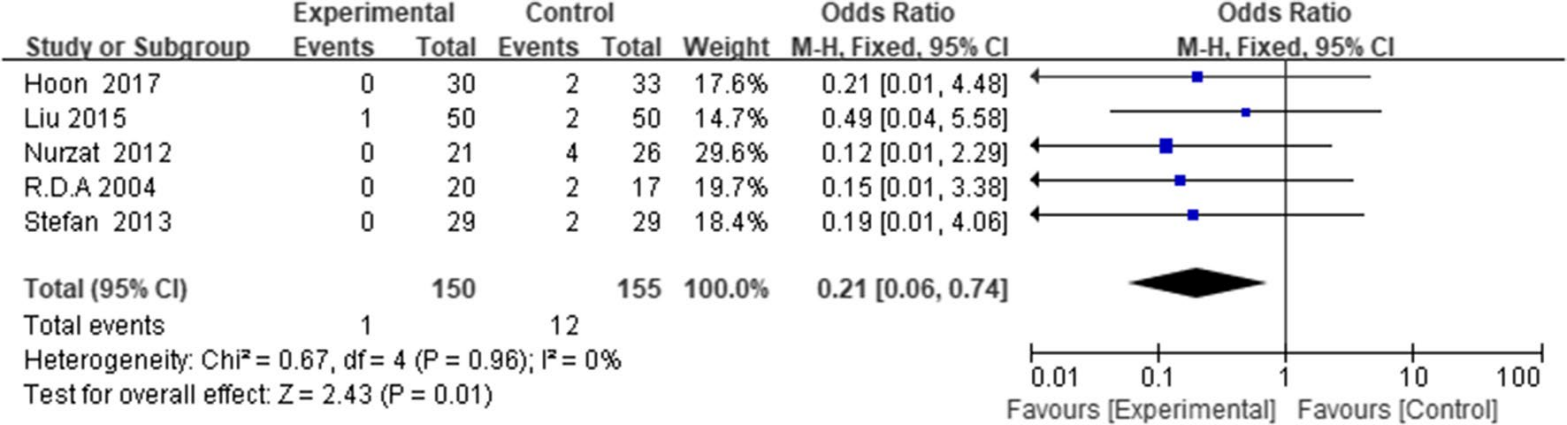
Forest plot of complications

Five studies [12, 18, 19, 23, 29] reported postoperative complications. The complications reported in the included studies mainly included tibial tuberosity fracture, wound infection, and delayed union or nonunion, all of which could be resolved by routine non-surgical treatment. The results suggested that DTO was associated with a greater number of complications (WMD = 0.21, 95% CI = (0.06 to 0.74), *p* = 0.01,* I*^*2*^ = 0%) (Fig. 9). In the PTO group, only one patient developed a superficial infection. In the DTO group, 12 patients experienced complications: one had deep vein thrombosis, one developed surgical site infection, two patients had delayed wound healing, one had screw dislocation, three patients had tubercle fracture, and four patients were diagnosed with patellar fractures.

## Discussion

In this meta-analysis, a total of 15 studies with 910 participants were included to compare whether PTO was superior to DTO in the treatment of medial KOA. The following conclusions were drawn: ①Compared with DTO, PTO was more likely to result in postoperative patella baja, and increase postoperative PTS. ②Compared with PTO, DTO had a higher incidence of postoperative complications. ③The HSS knee score was not different between the two groups. ④Both PTO and DTO increased the mMPTA and FTA, and the extent of increase was not significantly different between the two groups.

To our knowledge, proponents in the field of orthopedic surgery have promoted OWHTO as a common knee surgery since it can reduce the pressure on the medial compartment of the knee joint by correcting the limb alignment, thereby slowing down the progress of the disease, reducing pain, improving symptoms, and avoiding TKA [30–32]. However, OWHTO is associated with several complications, such as the change in PH, increase in PTS, bone nonunion, and loss of correction angle, among others [33, 34]. OWHTO is classified into PTO and DTO. Theoretically, PTO has fewer complications and is more conducive to healing than DTO, which is consistent with our study conclusion. DTO does not lead to patella baja, and the osteotomy line is located at the lower 1/3 of the tibial tubercle which have little affect the original morphology and biomechanics of the patellofemoral joint, and may be more beneficial for long-term follow-up. For medial compartment osteoarthritis and varus knee, DTO has less impact on future TKA [46].

The decrease of PH in PTO may cause patella baja and increased PTS in the sagittal plane [4, 35, 36]. In PTO, the osteotomy surface is located on the tibial tubercle. In the process of correction, the tibial tubercle can shift laterally and distally, inevitably leading to a drop in the patellar position. The greater the degree of correction, the more obvious the change in patellar position. This explains the occurrence of postoperative patella baja [12, 37]. In addition, the occurrence of patella baja can seriously affect the joint range of motion and increase the difficulty of TKA in the future [38, 39]. Theoretically, compared with PTO, the osteotomy surface in DTO is under the tibial tubercle; thus, the structure or position of the proximal tibia remains unchanged during the orthopedic process. In view of this advantage, DTO is increasingly being favored by orthopedic surgeons. Recently, several studies [20, 21, 23] have confirmed that there is little change in the patellar position after DTO, consistent with the results of our meta-analysis. Therefore, when the degree of correction is large, we recommend DTO to avoid postoperative patella baja.

The tension in the cruciate ligament and the pressure on the tibial plateau are both influenced by PTS. When the PTS angle is too large, it causes anterior and posterior instability and leads to ACL degeneration [40]. On the contrary, when the PTS angle is too small, the patient's knee flexion-gap increases, resulting in knee instability [41]. Therefore, the interference of PTS needs to be reduced in all knee surgeries. As per reports, PTS increase after OWHTO [42, 43] may be due to the unique anatomical features of the proximal tibia [37, 44]. PTS may also be increased when the osteotomy plane is not level with the sagittal tibial articular plane, the osteotomy gap is wide in the front and narrow at the back, and in cases of hinge fracture [45]. This study found that PTS differed between PTO and DTO, with the PTS in PTO being significantly higher. This may be because the osteotomy surface in PTO is located at the tibial junction. During the orthopedic process, the patellar tendon is subjected to traction during the osteotomy, which is more likely to cause an imbalance between the anterior and posterior osteotomy. However, there is no traction of the patellar tendon in DTO, which was found to have little impact on PTS in this study.

Theoretically, the greater the angle of PTO correction, the easier it is for patella baja to occur. In our study, this was reaffirmed by the lack of difference found between PTO and DTO in the degree of correction (MPTA), thereby excluding the influence of the degree of correction on the position of patella. The meta-analysis also found no difference in the HSS knee function score in the short term between PTO and DTO. Although, when compared with DTO, PTO can lead to a decline in patellar position and an increase in PTS, both of them can significantly improve knee function.

The most common complications after OWHTO are tibial plateau fracture and infrapatellar nerve injury, in addition to complications of conventional lower extremity surgery, such as infections, deep vein thrombosis, and delayed healing of the osteotomy surface [46]. In PTO, the patellar tendon works as a tension band for the anterior flange, which provides the compressive force to the coronal osteotomy plane during knee flexion. On the other hand, in DTO, the patellar tendon can detach the flange during knee flexion. Moreover, we should be paid great attention to during the DTO to avoid excessive penetration of the posterior cortex, which involves a potential risk of popliteal neurovascular bundle injury [47]. Another point to note in surgery is that the tibial tubercle is disadvantageous for bone healing due to its high proportion of cortical bone. Furthermore, screw fixation can be a cause of infection because of the skin irritation by the screw head, which may result in delayed union. The thicker flange for preventing the tuberosity fracture may make the thinner hinge, which can be the source of hinge fracture [48]. The above points are consistent with the results of this study, in that compared with PTO, the incidence of complications with DTO was higher.

Our study had several limitations. First, the studies included were retrospective in design, and the difference in relevant statistical indicators might have been caused by varying skills and expertise of surgeons, or differences in patients themselves, resulting in statistical heterogeneity. Second, the number of included cases was small. Third, most of the included studies had short periods of follow-up and did not reflect differences in long-term outcomes. Finally, the correlation between preoperative and postoperative HSS functional scores, PTS, and tibiofemoral angle changes were not assessed, and need further study.

## Conclusions

In this meta-analysis, we found that in comparison to DTO, PH was more likely to decrease and PTS was more likely to increase in PTO. Compared with PTO, DTO was associated with a greater frequency of complications. There were some differences in knee-joint parameters between PTO and DTO; however, their early clinical effects were similar and no differences were observed. We hope that our study provides further clinical evidence for both clinicians and patients making decisions regarding the operation method of OWHTO for medial compartmental OA.

## Data Availability

The datasets used and analysed during the current study are available from the corresponding author on reasonable request.

## Abbreviations

OWHTO: Open-wedge high tibial osteotomy
KOA: Knee osteoarthritis
TKA: Total knee arthroplasty
PTO: Proximal tibial tubercle osteotomy
DTO: Distal tibial tubercle osteotomy
ISI: Insall-Salvati index
CDI: Caton-Deschamps index
BPI: Blackburne-Peel index
PTS: Posterior tibial slope
HSS: Hospital for Special Surgery
